# “Found Performance”: Towards a Musical Methodology for Exploring the Aesthetics of Care

**DOI:** 10.3390/healthcare5030059

**Published:** 2017-09-18

**Authors:** Stuart Wood

**Affiliations:** Research and Knowledge Exchange, Guildhall School of Music and Drama, London EC2Y 8DT, UK; stuart.wood@gsmd.ac.uk

**Keywords:** healthcare, medical humanities, music therapy, performance

## Abstract

Concepts of performance in fine art reflect key processes in music therapy. Music therapy enables practitioners to reframe patients as performers, producing new meanings around the clinical knowledge attached to medical histories and constructs. In this paper, music therapy practices are considered in the wider context of art history, with reference to allied theories from social research. Tracing a century in art that has revised the performativity of found objects (starting with Duchamp’s “Fountain”), and of found sound (crystallised by Cage’s 4′ 33) this paper proposes that music therapy might be a pioneer methodology of “found performance”. Examples from music therapy and contemporary socially engaged art practices are brought as potential links between artistic methodologies and medical humanities research, with specific reference to notions of Aesthetics of Care.

*What* *is happening in this century, whether you accept it or not, is that more and more there is no gap between art and life...*

*Formerly,* *one was accustomed to thinking of art as something better organized than life that could be used as an escape from life. The changes that have taken place in this century, however, are such that art is not an escape from life, but rather an introduction to it…*

*And* *that's why I keep reiterating that we're working with our minds. What we're trying to do is to get them open so that we don't see things as being ugly, or beautiful, but we see them just as they are…*

John Cage

## 1. Definitions and Framework

This paper seeks to reframe music therapy (It is anticipated that readers may draw their own analogies with allied arts in health practices. For clarity, the discussion here is kept within the terms of music therapy.) within art history, rather than solely within healthcare (This term here refers also to discourses associated with “medicine”, “care”, and “social care”.) discourses, and to consider the implications of this reframing for healthcare practice and scholarship. It co-opts the fine art discourse of “performance”: not the performance of the stage or concert hall, but the performance of everyday life, framed as art, as popularised over the latter part of the twentieth century and emerging out of found object and found sound movements [1,2,3]. The term “performance” can be approached via a number of pathways, most literally via drama theory but also in relation to gender, philosophy, and ethnography (in theatre [4]; in relation to gender [5]; in the theatrical turn in philosophy [6,7]; and in ethnography [8,9] for example). In healthcare, “performance” involves an ethical and naturalistic exploration of the belief that health is not a static state, but rather an ongoing social co-production [10]. Music therapy [11] (the author in this paper acknowledges his own tradition of Creative Music Therapy as informing aspects of the discussion here, particularly with reference to the possibility of social intervention) is often situated in this approach to healthcare, in many cases modelling a practical way of perceiving and relating to people within healthcare services. The centrality within healthcare of an aesthetic process such as music-making invites reappraisal of the healthcare relationship and of the systems that maintain it. By encountering patients in their own performance of health as if they were already in an aesthetic mode, the music therapist is able to access more of their ability or personhood, and re-present this enhanced person to the healthcare audience. It is suggested here that “finding performance” in the everyday may open up a general application for aesthetic methodologies in medical humanities. It is argued that music therapy could be a valuable example of “found performance”. A vignette from music therapy practice is offered as an entry point to the discussion.

## 2. A First-Person Vignette from Music Therapy

Cheryl (names have been changed, in accordance with consent agreement to discuss this experience in professional situations) was in her mid 50s and living with MS when she had a serious subarachnoid haemorrhage. That is, she had a bleed in the fluid space that covers her brain. It led to a devastating brain injury. So, from having been a loving mother and a holistic practitioner, she became a patient in a hospital. Not only that, but she was someone who came over very badly under the normal battery of acute rehabilitative tests. She came to her care home with a very bleak prognosis: low awareness, nil meaningful response, keep warm and well. Care staff do not often take “nil” for an answer. We wanted to know for ourselves how Cheryl related to the world, and how we might get close. Our way of finding out was less objective and less standardized than medical rehabilitation tests are, and we had the flexibility to try many methods of connection during this time.

On my first encounter with Cheryl, I entered her room to see her in a reclining chair, looking pale and still, both arms splinted and flexed, her eyes not moving. I read in her rehabilitation assessments that she had no eye tracking towards motion or sound, no apparent shock response, and no reliably communicative vocal sounds. What she could do, clearly, was breathe. In line with my tradition in Creative Music Therapy [12] (Creative Music Therapy is an approach in which the music therapist seeks to make contact with the client via musical components or structures that match the client’s state at the moment of encounter.) I resolved to match her breathing with my breathing, and to enhance musically what she could do naturally. When I had slowed my breathing to her pace, I made a simple vocal sound on her out-breath, responding musically to her body’s natural processes. I did this wondering if my own voice, when timed with her movements, might trigger activity in her own body. I saw subtle changes: more flushing, more involuntary movement, more saliva. I changed my vocal tone, changing pitch, length, and volume to reflect these micro-changes in her. This could be perceived as a speculative or enquiring phase, in which I made musical choices in the belief [13,14] that it might lead to further arousal, forming the basis for interpersonal connection. Over ten minutes this loop of changes in my voice and her vitality continued, and my own vocalising grew into short rhythmic phrases. Then Cheryl breathed in an enormous convulsive breath and as she exhaled, a sound happened.

Was this sound responsive, or accidental? Certainly, it was enough for me to see Cheryl again. Later the same week I returned with a keyboard, intending to have the resources of harmony and texture at my musical fingertips if they were required. As I met her natural vocal sounds with carefully chosen keyboard harmonies, I was shocked by how her will and intention seemed to appear within her now more frequent sounds. In a sustained period of sequential out-breaths and turn-taking fragments, Cheryl toned her out-breaths, their pitch varying usually around the interval of the minor third. Over subsequent weeks, I explored new dimensions of interaction with Cheryl, finding shades of expressive vocalisation in these delicate musical moments. Through music I was able to match myself closely to her material presence, but at the same time to imbue that materiality with a semiotic component that potentially could be processed by Cheryl, transforming her everyday life into a shared performance.

## 3. Music Therapy as a Knowledge Practice

Voice is a form of knowing by doing (informed by speech-act theory [15] and more recent texts on epistemologies of praxis, [16]). The knowledge gained with Cheryl was derived from an informed technical enquiry, in which a practical experience of co-production led to tentative clinical insights. In its knowledge practice, music therapy enables practitioners to reframe patients as performers, the method of knowledge creation (usually musical improvisation) being simultaneously the knowledge that is created. The sounds produced in this practice are performative of new social relations. This concept of performativity in healthcare has roots in the influential music therapy writing of Aldridge [10,17], and Ansdell [18].

Arguably, music therapy presents a knowledge practice that engages with pre-existing conditions in such a way that their inherent aesthetic dimension is foregrounded. This is a uniquely artistic practice set within the most standard healthcare settings, and yet its methodological approach is relatively novel in medical humanities and allied scholarship (notable exceptions such as Ansdell & DeNora [19,20], address this gap). This paper seeks to consider a bridge between music therapy and medical humanities scholarship. That bridge is a new concept of “found performance”, drawing from the wider context of art history. Within a century that has seen performativity revise aesthetic relations through found objects (starting with the “readymade” of Duchamp’s famous urinal), and found sound (crystallised by Cage’s 4′ 33), this paper proposes a third category of the “found”, located in the social realm. It suggests that the medical humanities might be a pioneer site of “found performance”.

## 4. Found Object/Found Sound/Found Performance

In 1917, Marcel Duchamp notoriously submitted to exhibition an inverted standard porcelain urinal, signed with a pseudonym and named “Fountain”. His immediate intention was to challenge the assumptions of the board of New York’s Society of Independent Artists, and on this score, the work was successful. The board voted to contravene their own constitutional tenet of accepting all artists’ submissions, and rejected it.

Viewed by many to be anti-art, “Fountain” can alternatively be seen as a direct development of traditional Kantian aesthetics. In Kant’s Critique of Judgment, a defining feature of aesthetic judgment is “disinterestedness”. An aesthetic judgment:

...is made by some part of the self unlike what we normally think of as Subjectivity. Yet, neither can the judgment be objective, in the sense of rational or cognitive. This is because the object of aesthetic judgment is one that eludes conceptual definition and cognitive clarity. It is the focus of an opaque, if suggestive, sensory experience. And it is this opacity that stimulates the free play of imagination and understanding. In concert, these two faculties search for and find analogies, associations, formal rhymes and rhythms.

[2]

Seemingly, “Fountain” emerges from an attempt to produce a “disinterested” gaze in the viewer, explaining why Duchamp’s subsequent work with what became called “readymades” (The term “readymade” was first used by French artist Marcel Duchamp (1913) to describe the works of art he created from pre-existing manufactured objects. It has since often been applied more generally to artworks by other artists made in this way. It tends to denote a neutral gaze in the artist, unlike “found objects” which often refer to processes evoking Freudian interpretations and signalling a more personal object relation.) was characterised by his choice of objects that generated in him a feeling of neutrality. Perhaps in the readymade and later in the “found object” associated still with living artists such as Richard Wentworth [21] we have a gaze which cannot be categorised nor fully fixed. It is into an aesthetic gaze that the found object has delivered us. Opening a space between subjectivity and objectivity, between knowing and not-knowing, the aesthetic gaze recalls a third state: of disinterestedness, non-knowing (As distinct from not-knowing, which is located still within the paradigm of positivist knowledge, only as its negative. By contrast, non-knowing sits within an aesthetic not-needing-to-know. This gaze allows the object to come towards the viewer, actively), and non-attachment. In this state, the object in Heidegger’s terms “sets itself to work” (paraphrased from Heidegger [22]).

Beyond the gaze, the found object questions notions of commodification, the mark of the creator, the meaning of reproduction, and standardisation. Authorship and the mark of the creator were of particular interest to John Cage, a friend and colleague of Duchamp. It is taken in popular culture that Cage’s “urinal” moment was his framing of silence in his piece 4′ 33. In this work, a musician plays nothing for three periods of fixed duration. Instead of hearing sounds from a piano, the concert audience is invited to notice the many other sounds that surround them in the auditorium. Prepared by the context to listen musically, they are led into a mode of non-knowing that makes meaningful order out of what they might otherwise have considered random or not noticed at all. Cage’s aesthetic extends beyond the framing of sounds in a concert hall:

...I was with de Kooning once in a restaurant and he said, “If I put a frame around these bread crumbs, that isn’t art”. And what I’m saying is that it is. He was saying that it wasn’t because he connects art with his activity—he connects with himself as an artist whereas I would want art to slip out of us into the world in which we live.

[3]

Subsequent works in the musical canon (Berberian’s Stripsody, Reich’s City Life, and Blythe & Cork’s London Road, for example. Messiaen’s explorations into transcriptions of bird song are also compelling, despite his instinct for cataloguing.) have proceeded to explore this framing of the everyday world via a mode of musical listening. This mode is characterised by an aesthetic quality of non-knowing, of resisting reduction into either a subjective or objective modality, and of resisting categorisation. Within this mode, the object or now the sound is allowed to emerge and meet the gaze or ear. If the viewer or listener is prepared (by approach or circumstance) to meet it without judgment or fixing in categories, the object or sound performs itself in collaboration with them.

A third material of performance which was popularised in the 1960s by the Fluxus artists brings aesthetic judgment even closer to home for the healthcare researcher:

In Performance art, familiar daily activities of people in industrial societies are presented in a rather un-sophisticated theatrical way with every-day objects as props, and individual demonstrations of an artist’s ego are supposed to be avoided.

[1]

Echoing the neutrality and disinterestedness of Duchamp’s readymades, the anti-ego processes of Cage’s musical compositions, and the primacy of the everyday, Performance art seeks to present actions and scenarios from human social interaction for aesthetic judgment. A recent performance at New York’s Museum of Modern Art (The Artist Is Present, 2010) saw artist Marina Abramovic sitting impassive in the centre of a large gallery space for a total of 700 hours, meeting the gaze of almost 1400 people in turn. One of Abramovic’s earlier works, “Cleaning the Mirror”, presents her on film slowly scrubbing clean a human skeleton. The piece “serves to expose that which is most personal to the individual; a restated persona viewed as in a dream both externally and subjectively at the same time. While watching your innermost physical structure being picked painfully clean, you feel a sense of shocked recognition similar to seeing an X-ray” [23]. This is potentially more familiar territory for the medical humanities scholar, and yet its connection with the history of aesthetic judgment, and the contemporary role of the artist in healthcare may be a new kind of constellation. What does it add to medical humanities research for us to employ the performative role of objects, sound, and daily life?

Still more recently, in 2016 artist Jack Tan entered a work for the Singapore Biennale entitled Voices from the Courts [24]. In this work, he presented the daily activity of the Singapore State Courts via a performative artistic methodology. Here he produced graphic scores out of paintings derived from close listening to legal cases in action, exhibiting both the scores and their musical interpretations as a “found performance” of litigation in Singapore. Approaching the social as a material for aesthetic judgment is an established method in Performance Art. Tan’s work (and that of other artists such as Clifford Owens for example) seeks to challenge the authorship and elitism inherent in artist performances. Its techniques echo Cage’s chance procedures and the tradition of graphic scores to recalibrate the balance of power between material, artist, and viewer or listener. In this emerging artistic methodology, we find resonances with socially-oriented research strategies which hold similar values and which contend with similar subject matter.

## 5. Towards an Aesthetics of Care

The emerging argument here is that knowledge practices in music therapy offer a gaze (or a listening) that meets the participant as they emerge in performance. There is a parallel to this in the practice of Performance art, in which scenes from daily life are framed according to the aesthetic gaze, and transformed in the social relations they produce. The common mechanism between music therapy and the current social turn in Performance art could be described as a methodology of “found performance”. This methodology has a correlation in ethnographic practices and research strategies based in social justice, ethics, and care. If aesthetic knowledge practices such as music therapy can be understood in terms of care, perhaps care can be understood in aesthetic terms.

It is not new to frame care interactions as musical. Prominent psychiatrist Daniel Stern considered this a basic of adult–infant interaction:

...why is it necessary to add a new term for certain forms of human experience? It is necessary because many qualities of feeling that occur do not fit into our existing lexicon or taxonomy of affects. These elusive qualities are better captured by dynamic, kinetic terms, such as “surging,” “fading away,” “fleeting,” “explosive,” crescendo,” “decrescendo,” “bursting,” “drawn out,” and so on.

[25]

Neither is it novel to propose an aesthetic lens for analysing the social, as DeNora shows with reference to Adorno’s demonstration of “...how music’s formal properties evinced modes of praxis that in turn were related to, and could inculcate modes of, consciousness…” [26]. Goffman’s explorations of dramaturgy as a metaphor in sociology [27] are another notable example. This notion was taken up more recently by Spatz, whose exploration of praxis-based knowledge suggests that “we come to know ourselves, others, and the material world through the myriad pathways of technique...” [16]. Theorists such as Stuart-Fisher [28] and Thompson [29] have developed parallel explorations into how performativity intersects with knowledge, meaning, and action within a care frame. In a recent medical paper, Senior [30] proposed viewing GP consultations as performance art. Music therapy and the arts in health sector [18,31], are also starting to engage with health and social care from a performative perspective. As yet however, there has been scant communication between these discourses.

Is it possible that a concept of “found performance” presents a unique and timely intersectional opportunity for the medical humanities? An aesthetic methodology may present an apt response to current issues of commodification, evidence-based practice, care-based ethics, and relationship-based care (this argument has a natural fit with the established fields of Arts-based Research and Practice as Research. It is proposed in isolation here in order to explore a uniquely intersectional perspective on aesthetic judgment and non-knowing. It is suggested that the concept could prove useful in numerous research paradigms, not least that of Medical Humanities). This paper suggests that a concept of “found performance” could make visible numerous aspects of healthcare practice that standard modes of research or knowledge practices cannot.

## 6. A Found Performance in Room 34

The room number 34 was invented for the purposes of the paper.

*On* *my first encounter with Cheryl I entered her room to see her in a reclining chair, looking pale and still, both arms splinted and flexed, her eyes not moving...*

Understanding how Cheryl’s condition is articulated in her neurological assessment, and honouring the diagnostic and treatment processes that have kept her alive, restoring her after profound trauma, I refrain in this moment from occupying the same territory. I am aware of her medical history, but am more alive to her creative potential. My gaze in the doorway of Room 34 is not seeking to name her symptoms nor yet to articulate what I have yet to name, but in Iverson’s words, to stimulate “the free play of imagination and understanding search for and find analogies, associations, formal rhymes and rhythms” [2]. This is neither to objectify nor reduce Cheryl to the status of the inanimate, but by contrast to do what this gaze does: to value and make sacred, and to set her to work.

How is this achieved, here in Room 34? By putting an alternative frame around us. In the silence I hear Cage saying, “...*what is happening in this century, whether you accept it or not, is that more and more there is no gap between art and life…*” [3]. If I hear every sound as if it is music, I hear not only its tone and timing, but I hear its place amongst other sounds, and I impute into it a semiotic or expressive intention. As a music therapist, my best method is to join in musically. This is both to produce sounds in response, but also to think alongside, compositionally. In this way, I also—in the tradition of Messiaen, Berberian and Reich—give the person that I have found here a new value through close compositional attention to their unique sounds. Whilst I claim that her sounds in the first instance seem to emerge in response to my actions, those actions are part of a non-knowing encounter in which I simply hear what is coming towards me in the silence. Neither I, nor music therapy, created those sounds. We might say we found them. My response to this sound is to allow it to emerge along the pathway of harmony and timing that I have set in motion. Avoiding the snare of believing that my sole actions are a causal treatment for specific symptoms, (this notion invokes an ecological perspective on the foundational stance of music therapy [32], and should not be taken to suggest that music therapy is not valuable as part of multidisciplinary approaches to medical treatment. In many cases, it is [13]). I invoke the common wisdom of the performance artist that, “individual demonstrations of an artist’s ego are supposed to be avoided” [1]. That is, if we assume here that the artist is me.

More likely, the artist is every person who forms the scene currently playing out in Room 34. Subsequently, what I learn with Cheryl can be shared professionally with all the dramatis personae in the “found performance” of the care home. We may note how this knowledge creates co-performers in resident, therapy staff, housekeeping, maintenance, nursing, and care staff [31], and how sensitive they may become to their own sound production. To avoid attending only to the acoustic environment, we may also make use of existing knowledge around the musicality of interpersonal care interactions [25,33], and refine that knowledge in the context of social care. Musical techniques from the canon of found sound and verbatim musical transcription have more yet to offer, and would help steer some scholarship away from a reliance on the production of affect or spectacle. We may also recognise that it is an interdisciplinary task to mark how an aesthetic sensitivity in the workplace of care can recalibrate the balance of power between resident and staff member; to perform differently the roles of viewer, viewed, listener, listened to, carer, and cared for. Further discussion on the validity of performance ethnographies, a voluntary code of practice for socially engaged artists, and further opportunities for academic exchange would all be viable paths of development. Interdisciplinary research into the resonances of medical ethics [34], patient safety [35], and the healthy environment [36] would translate the aesthetic basis of music therapy to the central concerns of medical humanities.

## 7. Conclusions

The aim of this paper was to introduce the notion of ‘found performance’, and to explore its potential as a methodology for understanding healthcare. Music therapy was used as an example from healthcare in which the concept of performance is already established, and a vignette from practice gave grounds for discussion. It was suggested that epistemologies of technique and praxis are central to music therapy, and that this might be exemplary of wider healthcare and medical practice. The craft of healthcare, or its inherent ‘knowing by doing’ might helpfully be understood through an aesthetic lens. This aesthetic lens would ascribe a more active role to the traditional objects (materials and equipment) or participants (patients and users) of healthcare. It would suggest that those objects and participants co-construct the meaning and provision of healthcare. 

The history of art offers a useful framework for this through the twentieth century threads of found object, readymade, found sound and performance. Iconic works such as Duchamps’ Fountain, Cage’s 4’33 and Abramovic’s The Artist Is Present, were brought as examples of how the aesthetic gaze has turned towards what ethnographers would call ‘everyday life’. To frame everyday life as performative, as already art, is a staple of music therapy practice but a notion little explored in wider healthcare. This stance would instigate an ecological gaze towards not only the people who participate in healthcare treatment, but also to the practices, knowledges and materials that co-construct healthcare systems. An aesthetics of care would build on this re-framed ecology with reference to ongoing issues in healthcare and the medical humanities such as medical ethics, patient safety, and the healthy environment. The notion of ‘found performance’ may be a creative entry point to developing an aesthetic methodology within medical or healthcare research. 
